# Usefulness of a new DUV-LED device for the control of infection by *Escherichia coli, Staphylococcus aureus*, mycobacteria and spore-forming bacteria

**DOI:** 10.3389/fpubh.2022.1053729

**Published:** 2022-12-05

**Authors:** Hiroko Inagaki, Yoshitaka Goto, Hironobu Sugiyama, Akatsuki Saito, Tamaki Okabayashi, Kyosuke Watanabe, Shouichi Fujimoto

**Affiliations:** ^1^M&N Collaboration Research Laboratory, Department of Medical Environment Innovation, Faculty of Medicine, University of Miyazaki, Miyazaki, Japan; ^2^Nikkiso Co., Ltd., Tokyo, Japan; ^3^Department of Veterinary Science, Faculty of Agriculture, University of Miyazaki, Miyazaki, Japan; ^4^Center for Animal Disease Control, University of Miyazaki, Miyazaki, Japan

**Keywords:** deep-UV LED, microorganism, germicidal effect, floor sterilization, LED device

## Abstract

Reliable disinfection and sterilization technologies are needed to deal with the various infectious diseases spreading around the world. Furthermore, bacteria that are difficult to eliminate by ordinary disinfection are also a problem in the medical environment. We examined the germicidal effect of a newly developed deep-ultraviolet light-emitting diode (DUV-LED) prototype device (wavelength of 280 ± 5 nm; power of 0.9 to 1.4 mW/cm^2^) for floor sterilization against *Escherichia coli (E. coli), Staphylococcus aureus (S. aureus), Mycobacterium gordonae (M. gordonae)*, and *Bacillus subtilis (B. subtilis)*. This prototype device is equipped with highly practical DUV-LEDs with a high output efficiency and a long life, and was designed with consideration of the irradiation distance and the angle of the DUV-LEDs to provide a uniform irradiation rate. We found a statistically significant reduction of ≥90% in the infectious titers of both *E. coli* and *S. aureus* after irradiation for 2 s. Although acid-fast bacilli and spore-type bacilli are generally thought to be resistant to UV light irradiation compared to general bacteria, the acid-fast bacillus *M. gordonae* was inactivated after irradiation for 10 s, and spore-type cells of the bacillus *B. subtilis* were inactivated by ≥90% after irradiation for 30 s. We also found that the effects were cumulative when irradiation was performed at intervals. In the future, the usefulness of this device as an infection control measure will be evaluated in daily medical practice.

## Introduction

Medical facilities have long been committed to infection control, and drug-resistant bacteria are often a problem. However, the existence of bacteria that cannot be removed easily by ordinary disinfection is becoming a problem in the medical field. The recent coronavirus disease 2019 pandemic has created a need for more effective and sustainable infection control measures. Thus, the development of reliable disinfection and sterilization technologies are needed for dealing with the various infectious diseases spreading around the world. The transmission of infectious microorganisms to humans occurs mainly through direct contact and airborne particles. Microorganisms that fall and accumulate on floor surfaces may also re-scatter, form droplets, and cause airborne and contact infections.

**Figure 1:**
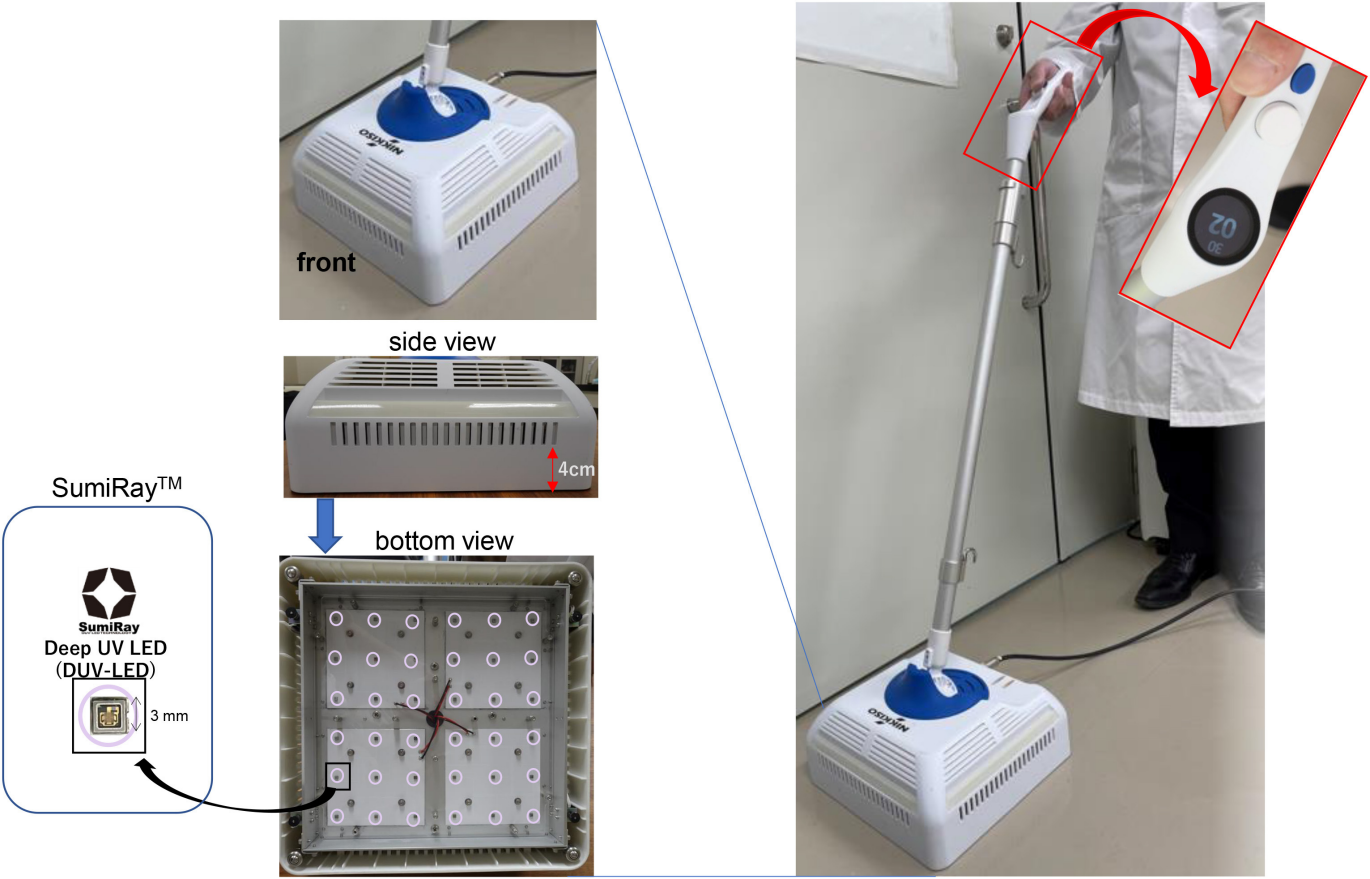
Picture of newly developed DUV-LED-equipped device.

Deep-ultraviolet (DUV) light-emitting diode (LED) irradiation is known to inactivate various microorganisms (1–6), and we have already reported the efficacy of DUV-LED irradiation in inactivating severe acute respiratory syndrome coronavirus 2 (7, 8). UV-C (wavelength of 100–280 nm) is considered to be the most effective germicidal region of the UV spectrum as it causes the formation of photoproducts in DNA and RNA (9). These pyrimidine dimers interrupt the transcription, translation, and replication of DNA and RNA, eventually leading to the death of the microorganism (10). Therefore, DUV is expected to be useful as a countermeasure against nosocomial infections, regardless of sensitivity for antibiotics and disinfectants. In this study, a prototype of a floor sterilizer equipped with UV-LEDs (Figure 1) was created for everyday use, and we investigated the inactivating effect of this prototype on *Escherichia coli (E. coli), Staphylococcus aureus (S. aureus), Mycobacterium gordonae (M. gordonae)*, and *Bacillus subtilis (B. subtilis)*.

## Newly developed DUV-LED device for floor sterilization

The newly developed DUV-LED device for floor sterilization was designed and developed in collaboration with Nikkiso Co., Ltd. (Tokyo, Japan) and Chiba University (Chiba, Japan), and was manufactured by Saney Seiko Inc. (Saitama, Japan). The device consisted of a main body, a pole, and a handle (Figure 1). The main body was designed to be 250 × 250 mm in size with 36 DUV-LED chips on the underside. The DUV-LED chips (*SumiRay*™), which generate a narrow-range wavelength (280 ± 5 nm), were obtained from Nikkiso Co. Ltd. This wavelength was selected, during the LED development phase for practicality, in order to obtain the high output (radiation) power and long life of DUV-LEDs. The LED layout was designed based on optical analysis using LightTools software (Synopsys, Inc., Mountain View, CA, USA), and the irradiation distance to the floor surface was 40 mm. In addition, a resin with a transmittance of 80% (thickness of 15 mm) was used to protect against DUV exposure. The illuminance of DUV rays ranges from 0.9 to 1.4 mW/cm^2^, and the central area has the highest power. The irradiation time can be adjusted from 1 to 99 s, and the structure was designed with consideration for mobility.

## Germicidal effect of the newly developed DUV-LED device

The *E. coli* ATCC25922, *S. aureus* ATCC29213, *M. gordonae* ATCC14470, and *B. subtilis* ATCC6051-T strains were used as the test organisms. We chose these bacteria, because *E. coli* and *S. aureus* species are often found in medical facilities and in household environments, and *M. gordonae* and *B. subtilis* are known to be resistant to disinfectants and can cause opportunistic infections. The *E. coli, S. aureus*, and *B. subtilis* were grown aerobically on trypticase soy agar (TSA; Becton, Dickinson and Company, Franklin Lakes, NJ, USA) at 37°C. Single colonies were then inoculated into trypticase soy broth (TSB) and cultured overnight at 37°C. The culture was then divided into 1-ml aliquots, and stored frozen at −80°C until used for the tests. *M. gordonae* was also cultured in the same manner, with the exception that the cells were cultured aerobically in Middlebrook 7H9 Broth. For the UV irradiation test, the cryopreserved bacteria were rapidly thawed and diluted with fresh TBS to a concentration of 10^4^ to 10^5^ cells/ml. *M. gordonae* was cultured without shaking for several days, while the other bacteria were cultured with shaking for about 3 h before the bacterial solutions were diluted to a concentration of 1 × 10^4^ to 1 × 10^6^ cells /ml to prepare inoculum solutions. In addition, for *B. subtilis*, the bacterial solution was washed three times with 70% alcohol to obtain only cells of the spore type. Subsequently, 100 μl of each inoculum solution was applied to the surface of a TSA plate (solidified to a thickness of about 3 mm in a plastic Petri dish with a diameter of 9 cm), except for *M. gordonae*, which was applied to the surface of a Middlebrook 7H10 agar plate of the same size, and the inoculum was spread evenly with a spreader. These inoculated plates were then used for the irradiation test.

For the irradiation test with the DUV-LED floor sterilization prototype, each plate was irradiated for 1, 2, 3, or 5 s for *E. coli* and *S. aureus*, 5, 10, 15, or 20 s for *M. gordonae*, and 5, 15, 30, 60, or 90 s for *B. subtilis*. The effect of DUV-LED irradiation was evaluated by counting the number of colonies after incubating the plates for 18 to 24 h at 37°C, except for *M. gordonae*, which was counted after about 2–3 weeks, and comparing the number of colonies to that on the non-irradiated control plates. In addition, an interval irradiation test was also performed on *B. subtilis*, which was irradiated 2 or 3 times at intervals of 30 min.

**Figure 2:**
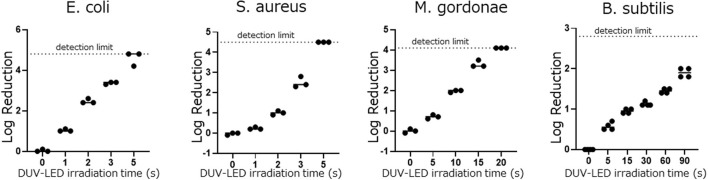
Differences in the log reduction after DUV-LED irradiation against the plates coated by each bacterial solution.

In the DUV-LED irradiation test, *E. coli and S. aureus* were reduced by >90% after 2 s of irradiation. Although acid-fast bacilli and spore-type bacilli are known to be resistant to UV irradiation, the acid-fast bacillus *M. gordonae* was reduced by 88.9% after 5 s of irradiation and by >90% after 10 s of irradiation, and the spore type of the bacillus *B. subtilis* was reduced by 74.5% after 5 s of irradiation and by >90% after 30 s of irradiation (Figure 2 and Table 1). The mixed vegetative type of *B. subtilis* that had not been washed with alcohol was more likely to be inactivated than the spore type. In addition, even when *B. subtilis* was irradiated 2 or 3 times at intervals of 30 min, the same microbial inactivating effect as that from cumulative continuous irradiation was seen (Supplementary Figure 1). We also confirmed that UV irradiation of the media alone had no significant effect on the subsequent microbial growth on the media.

**Table 1:** Differences in the infectious titer after DUV-LED irradiation against the plates coated by each bacterial solution.

***E. coli***	**0s**	**1s**	**2s**	**3s**	**5s**	
CFU/mL (±SD)	5.9 ± 1.2 × 10^4^	5.7 ± 0.9 × 10^3^	2.3 ± 0.5 × 10^2^	2.5 ± 0.2 × 10^1^	1.7 ± 2.1 × 10^0^	
log 10	4.8	3.8	2.4	1.4	0.2	
log reduction	–	1.0	2.4	3.4	4.5	
% reduction	–	90.3	99.6	>99.9	>99.9	
***S. aureus***	**0s**	**1s**	**2s**	**3s**	**5s**	
CFU/mL (±SD)	3.2 ± 0.7 × 10^4^	1.7 ± 0.2 × 10^4^	3.1 ± 0.3 × 10^2^	1.0 ± 0.4 × 10^2^	0	
log 10	4.5	4.2	2.5	2.0		
log reduction	–	0.3	2.0	2.5	>4.5	
% reduction	–	46.9	99.0	99.7	>99.9	
***M. gordonae***	**0s**	**5s**	**10s**	**15s**	**20s**	
CFU/mL (±SD)	1.0 ± 0.2 × 10^8^	2.2 ± 0.3 × 10^7^	1.3 ± 0.1 × 10^6^	6.7 ± 2.3 × 10^4^	0	
log 10	8.1	7.3	6.1	4.8		
log reduction	–	0.7	2.0	3.3	>4.1	
% reduction	–	79.5	98.9	99.9	>99.9	
***B. subtilis***	**0s**	**5s**	**15s**	**30s**	**60s**	**90s**
CFU/mL (±SD)	6.7 ± 0.2 × 10^6^	1.7 ± 0.4 × 10^6^	7.4 ± 0.5 × 10^5^	4.7 ± 0.6 × 10^5^	2.2 ± 0.2 × 10^5^	8.0 ± 1.7 × 10^4^
log 10	6.8	6.2	5.9	5.7	5.3	4.9
log reduction	–	0.6	1.2	1.5	1.9	2.3
% reduction	–	74.5	88.9	93.0	96.7	98.8

## Discussion

A variety of microorganisms are present in the hospital environment that are not easily removed by ordinary cleaning. Among them, some species of mycobacteria and of spore-forming bacteria can escape sterilization by disinfectants, and as a result, they infect immunocompromised hosts and cause serious infections although they have little effect on healthy individuals. These bacteria are problematic since they can spread easily, are resistant to ordinary antibiotics and disinfectants, and cause chronic infections that require long-term treatment (11, 12). The microbe-inactivating effects of DUV rays have been reported to be useful in preventing the spread of new infectious diseases (1–6). In particular, the *SumiRay*™ DUV-LED chips, which generate a narrow-range wavelength (280 ± 5 nm), installed in this prototype have some advantages in terms of practicality as they have a high output (radiation) power and a long lifetime. Among UV-C irradiators, *SumiRay*™ has been highly evaluated for its microbial-inactivating effect and high luminous efficiency (13). Hand-held far-UV irradiation (wavelength of 185–254 nm) devices have been reported to be effective for surface sterilization in a limited area (14, 15). However, unlike our prototype, these devices consist of UV lamps. In our prototype, the light source was changed from a mercury lamp to LEDs, which are advantageous as they are more environmentally friendly and can be instantaneous turned on and off. Sterilization of the floor surface can help to prevent the transmission of diseases as it inactivates microorganisms that have fallen and accumulated on the floor surface before they can re-scatter and cause droplet infections. However, even in healthy human adults, exposure to DUV-LEDs has unfavorable effects, such as conjunctivitis, keratitis, and dermatitis, which are concerns when using LEDs for sterilization. In this prototype, a sufficient number of UV monads are arranged by calculating the irradiation angle and irradiation distance so that the shadowed part can be covered as much as possible in the irradiation space, and it is structured so that UV exposure does not leak to the outside for our safety.

This study has some potential limitations. First, the experimental results were based on the assumption that microorganisms are directly irradiated on a flat floor surface free of organic materials. It may be possible to improve the effectiveness of this device by combining it with a vacuum cleaner that removes organic materials on the floor surface. Second, in this study, the inactivating effect of DUV-LED was evaluated only against bacteria in the culture solution. It has been reported by Mitchell et al. (16) that higher UV light doses are required to inactivate bacteria and viruses on hard surfaces than those in suspensions.

As environmental surfaces play an important role in the transmission of nosocomial pathogens, this device may be useful for floor sterilization in hospitals and medical facilities that require infection control. The prototype used in this experiment overcomes the problem of uneven irradiation that is seen with direct irradiation alone, and it enables high-power UV irradiation for the very rapid killing of pathogens as the DUV-LED light sources are arranged at optimal intervals. Another advantage of this DUV-LED-mounted sterilizer is that it can be used safely by anyone for a long period of time with minimal operation and maintenance costs.

In this study, the microbe-inactivating effect of this prototype device was evaluated by irradiating a bacterial suspension-coated Petri dish. After irradiation for 2 s, a ≥90% reduction in the infectious titers of both *E. coli* and *S. aureus* was observed; this short irradiation time is expected to be very useful considering the time it takes to clean floors. Even for bacterial species that have been reported to be resistant to DUV (17, 18), a ≥90% reduction was observed after 10 s of irradiation for *M. gordonae*, and after 30 s of irradiation for spore-type *B. subtilis*. Furthermore, irradiation at intervals showed the same microbial-inactivating effect as that from continuous irradiation, indicating that a sufficient inactivating effect can be obtained by divided irradiation. This is considered to be useful for the practical application of DUV-LEDs in automatic vacuum cleaners.

## Data availability statement

The original contributions presented in the study are included in the article/Supplementary material, further inquiries can be directed to the corresponding author/s.

## Author contributions

HI, HS, and YG conceived the study, wrote the manuscript, and conducted the experiments dealing with the bacteria. AS and TO managed and supported the experiments. HS and KW participated in the production of a prototype floor sterilizer equipped with UV-LED. SF contributed to the study design, study supervision, and manuscript revision. All authors have read and agreed to the published version of the manuscript.

## Funding

This study was supported in part by the Japan Society for the Promotion of Science (JSPS) KAKENHI Grant-in-Aid for Scientific Research (21H02361 to TO and AS), JSPS KAKENHI Grant-in-Aid for Scientific Research (19K06382 to AS), and a Grant for Joint Research Projects of the Research Institute for Microbial Diseases, Osaka University (to TO and AS).

## Conflict of interest

Authors HS and KW receive part of their salary from Nikkiso Co., Ltd., Tokyo, Japan. Nikkiso supplied the DUV-LED-equipped device for floor sterilization (prototype) for evaluation. Nikkiso Co., Ltd. had no role in the study design, data collection and analysis, decision to publish, or preparation of the manuscript. The remaining authors declare that the research was conducted in the absence of any commercial or financial relationships that could be construed as a potential conflict of interest.

## Publisher's note

All claims expressed in this article are solely those of the authors and do not necessarily represent those of their affiliated organizations, or those of the publisher, the editors and the reviewers. Any product that may be evaluated in this article, or claim that may be made by its manufacturer, is not guaranteed or endorsed by the publisher.

## Supplementary material

Supplementary Figure 1 Differences in the log reduction after DUV-LED irradiation against the plates coated by B. subtilis solution. •; • (circle) shows the change after DUV irradiation (5, 15, 30, 60, or 90 s, continuously) against the plate on which B. subtilis solution treated by alcohol to obtain only cells of the spore type. Δ; Δ (open triangle) shows the change after DUV irradiation (5, 15 s) against the plate on which B. subtilis solution untreated by alcohol. □; □ (open square) shows the change after twice (30 s x 2; 60 s, 60 s x 2; 120 s) or three times irradiations (30 s x 3; 90 s) with an interval of 30 min, respectively, against the plate on which B. subtilis solution treated by alcohol (for spore type).
