# Supplementary figures and images for: Usefulness of a new DUV-LED device for the control of infection by Escherichia coli, Staphylococcus aureus, mycobacteria and spore-forming bacteria
Source: Front Public Health. 2022 Dec 5;10:1053729. doi: 10.3389/fpubh.2022.1053729 (PMC9760979; doi:10.3389/fpubh.2022.1053729)

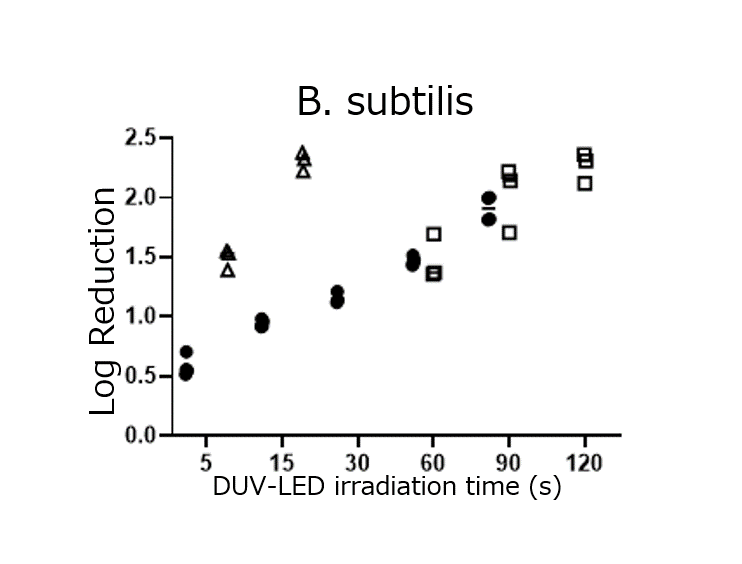

Supplement: Supplementary Figure 1 — Differences in the log reduction after DUV-LED irradiation against the plates coated by B. subtilis solution. •; • (circle) shows the change after DUV irradiation (5, 15, 30, 60, or 90 s, continuously) against the plate on which B. subtilis solution treated by alcohol to obtain only cells of the spore type. Δ; Δ (open triangle) shows the change after DUV irradiation (5, 15 s) against the plate on which B. subtilis solution untreated by alcohol. □; □ (open square) shows the change after twice (30 s x 2; 60 s, 60 s x 2; 120 s) or three times irradiations (30 s x 3; 90 s) with an interval of 30 min, respectively, against the plate on which B. subtilis solution treated by alcohol (for spore type). [file Image_1.TIFF]
